# Three-Stage Wiener-Process-Based Model for Remaining Useful Life Prediction of a Cutting Tool in High-Speed Milling

**DOI:** 10.3390/s22134763

**Published:** 2022-06-24

**Authors:** Weichao Liu, Wen-An Yang, Youpeng You

**Affiliations:** College of Mechanical and Electrical Engineering, Nanjing University of Aeronautics and Astronautics, Nanjing 210016, China; weichaoliu@nuaa.edu.cn (W.L.); youypeng@163.com (Y.Y.)

**Keywords:** remaining useful life prediction, Wiener process model, multi-stage division, cutting tool

## Abstract

Tool condition monitoring can be employed to ensure safe and full utilization of the cutting tool. Hence, remaining useful life (RUL) prediction of a cutting tool is an important issue for an effective high-speed milling process-monitoring system. However, it is difficult to establish a mechanism model for the life decreasing process owing to the different wear rates in various stages of cutting tool. This study proposes a three-stage Wiener-process-based degradation model for the cutting tool wear estimation and remaining useful life prediction. Tool wear stages classification and RUL prediction are jointly addressed in this work in order to take full advantage of Wiener process, as this three-stage Wiener process definitely constitutes to describe the degradation processes at different wear stages, based on which the overall useful life can be accurately obtained. The numerical results obtained using extensive experiment indicate that the proposed model can effectively predict the cutting tool’s remaining useful life. Empirical comparisons show that the proposed model performs better than existing models in predicting the cutting tool RUL.

## 1. Introduction

Cutting tool plays an important role in the machining system and its wear causes an increase in friction and heat generation in the machining process. Wear of cutting tools not only affects the quality of machined surface and the machining precision but also results in increasing machining cost. Moreover, unnecessary tool replacement that aims at preventing the decrease in surface quality will increase the downtime and machining cost in high-speed milling. Tool condition monitoring can be employed to ensure safe and full utilization of a cutting tool. Hence, the remaining useful life (RUL) prediction of a cutting tool is an important issue for an effective high-speed milling process-monitoring system.

In the past few decades, various studies have been devoted to predicting the cutting tool RUL in both academia research and industry. According to the preliminary research, these studies were developed based on the consideration of physics-based and data-driven models. The Taylor model [1] and the Forman crack growth model [2] are the typical physics-based models. Although these physics-based methods can accurately describe the degradation process of equipment and require less training data, it is very difficult if not impossible to build the exact physical model because of heavy dependence on the expert knowledge and the degradation law of equipment. Recently, intensive research has been conducted on the utilization of data-driven models, which was regarded as an effective tool for the RUL estimation of various degrading systems. Machine learning applications and statistical learning applications in the field of data-driven models have been reported to outperform the traditional physics-based methods. For instance, Benkedjouh et al. [3] extracted the features form vibration, AE and force signals and then utilized the support vector regression (SVR) to predict the tool RUL. Sun et al. [4] evaluated the RUL of a cutting tool based on the result of operational reliability assessment and back propagation neural network. Zhou et al. [5] used the long short-term memory (LSTM) model to solve complex correlation and memory accumulation effects and then established the RUL prediction model under different conditions. Babu et al. [6] proposed a convolutional neural network (CNN)-based regression approach, and then learned the features and estimated the RUL by the supervised feedback. Zhang et al. [7] proposed a deep belief network (DBN)-based multi-objective ensemble method for the RUL estimation in prognostics, and the experimental results demonstrated the superiority of the proposed method compared to the existing approaches. An et al. [8] developed a hybrid model by combining the CNN with the LSTM network and then predicted the RUL with sequence tool wear data. The proposed model proves to be efficient in tool wear evolution tracking and the RUL prediction accuracy reached up to 90%. Though the models in the above cited work proved to be effective in real applications, they ignore the correlation characteristics in the time-series wear data and cannot sufficiently model the tool wear process. In order to address this issue, the hidden Markov model (HMM) and its various variants, one of the most commonly used statistical learning techniques, have been comprehensively employed to describe the dynamic evolution of wear process due to the powerful ability in processing the time-series data. Geramifard et al. [9] proposed a physically segmented HMM to build the relationship between hidden stages with the real health stages of a cutting tool and the provided relationship was further used for diagnostics and prognostics. Following the study of [9], Geramifard et al. [10] proposed a multimodal HMM-based approach for tool wear monitoring. Yu et al. [11] developed a weighted HMM approach, which takes the wear rate as the hidden state. Then, they predicted the RUL and estimated the tool wear during tool operation. The experiment results show that their approach outperforms the conventional HMM approach. Zhu and Liu [12] utilized the hidden semi-Markov model (HSMM) to model the complex tool wear process and then estimated the tool wear and predicted the RUL of tool with a forward algorithm. The experiment results show that the proposed method leads to more accurate RUL prediction in high-speed milling.

Although these aforementioned methods appear to obtain not so bad RUL estimation results, it is still difficult to describe the nonmonotonic dynamic characteristics of system. Fortunately, the Wiener process has shown to be the optimal degradation process modeling tool for explaining the physical behavior of a dynamic system, due to its excellent mathematical properties and physical interpretations [13]. Ghorbani and Salahshoor [14] developed a degradation model for the engine fault of turbofan by combining a physics-based model and a Wiener process with positive drift. Li et al. [15] considered the various degradation processes of different units and developed a Wiener-process-model-based method for RUL prediction. For a complicated dynamic system, stochastic behavior is inevitable due to multiple sources of variability, which contribute to the uncertainty of the RUL estimation. Therefore, the effect of these uncertainties and different kinds of variability should be incorporated into a wear model to improve the accuracy of the RUL estimation. Tsai et al. [16] analyzed that some quality characteristics (QC) whose degradation over time can be related to the reliability of the product and established the degradation model by taking this variability into account. Sun et al. [17] modeled the tool wear process of a cutting tool with the Wiener process considering the measurement variability and then estimated the RUL of cutting tool.

The above research studies ignore the mechanism of degradation and regard it as an overall process. However, the degradation process (e.g., rotating machinery, lithium-lon batteries) exhibits multiple stages characteristics in practice due to the environmental working condition, internal materials and so on. Lim et al. [18] proposed a two-phase accelerated degradation test to efficiently derive product lifetime information in the development stage for new products, when reliability-related information is generally limited and there is limited sample availability. Zhang et al. [19] proposed a multi-phase stochastic degradation model based on the Wiener process and then used this method for the RUL prediction of lithium-lon batteries. Chen and Tsui [20] predicted the RUL of rotational bearings with a two-phase model, which characterizes and determines different stages of the degradation process. Wen et al. [21] proposed a flexible Bayesian multiple-phase modeling approach to characterize degradation signals for prognosis. Later, Wen et al. [22] extended the previous work and developed a multiple change-point Wiener process model for RUL prediction.

According to the literature review given above, the aim of this study is to develop a three-stage Wiener-process-based degradation model that can predict the tool’s remaining useful life in high-speed milling processes. First, the time domain, frequency domain and time–frequency domain features are extracted from raw sensor data (i.e., AE, cutting force, vibration), and then the stacked denoising autoencoder (SDAE) is utilized to automatically select the most relevant features. Second, tool wear stages and corresponding wear value are estimated with the extreme learning machine (ELM). Third, the degradation process of each wear stage is established based on Wiener process and the overall useful life is estimated with the stage RUL prediction. Additionally, multi-source variabilities (i.e., the inherent temporal variability of wear path, individual variability of machining condition or measurement variability of sensors) are considered and quantified in the modeling process of each stage, which helps describe the physical behavior of tool wear process more precisely and significantly improves the accuracy of RUL prediction.

## 2. Three-Stage Wiener-Process-Based Model

### 2.1. Motivation

**Figure 1:**
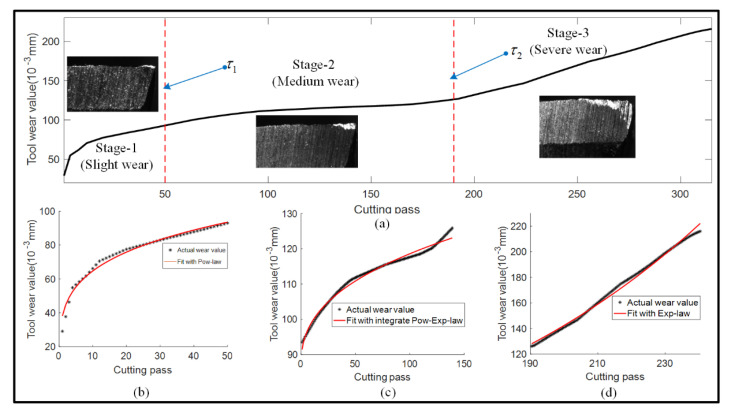
Three stages of tool wear in a cutting process. (**a**) The typical degradation process of a cutting tool; (**b**) Slight wear stage; (**c**) Medium stage; (**d**) Severe wear stage.

In most existing research studies, the degradation mechanism was assumed as stationary over the entire life of a cutting tool, which was characterized with a fixed model. However, the degradation process of a cutting tool exhibits multiple-stage characteristics in practice due to the environmental working condition, internal materials and so on. It can be seen from Figure 1a that a typical degradation process of a cutting tool can be divided into three stages depending on the wear rate, namely slight wear stage (Figure 1b), medium wear stage (Figure 1c) and severe wear stage (Figure 1d).

Motivated by this phenomena, three models were developed to characterize the features into different stages. It can be seen from Figure 1b that tool wear presents a sharp rate in the first stage, which can be fitted with power law. In the second stage depicted in Figure 1c, the wear rate becomes slow and can be covered with an integrated power–exponential law. When it turns into the third stage in Figure 1d, the wear rate gets faster and exponential law can be utilized to describe the degradation process. The basic law of these models can be formulated as follows:

(1) $$X\left(t\right)=\left\{\begin{array}{l} X_{1}\left(t\right)=a_{1}t^{b_{1}}+k_{1},\;\;\;\;\;t<\tau_{1}\\ X_{2}\left(t\right)=a_{2}t^{b_{2}}+c_{1}\exp^{d_{1}t}+k_{2},\;\tau_{1}\leq t\leq \tau_{2}\\ X_{3}\left(t\right)=c_{2}\exp^{d_{2}t}+k_{3},\;\;\;\;\;\;t>\tau_{2} \end{array}\right.$$

where t and X(t) denote the cutting pass and corresponding wear value, $a_{1}$, $b_{1}$, $k_{1}$, $a_{2}$, $b_{2}$, c, d, $k_{2}$, $a_{3}$, $b_{3}$, and $k_{3}$ are model parameters of different wear stages, $\tau_{1}$ and $\tau_{2}$ are the boundaries of the three stages.

### 2.2. Model Formulation

In practice, the wear process of a cutting tool usually exhibits a stochastic behavior with nonlinearity and multiple variability sources. The Wiener process has proved to be effective in characterizing the degradation signals due to its great mathematical properties. In order to quantify the multi-source variabilities (e.g., inherent temporal variability of wear path and individual variability of machining condition) and characterize the degradation process of each stage, a three-stage Wiener-process-based model considering multi-source variabilities was proposed in this subsection.

Let {S(t),t≥0} denote the degradation state of tool wear over time t with the initial value $S(0)=s_{0}$, and then the stochastic process can be expressed as follows:

(2) $$S(t)=S(0)+\int_{0}^{t}g(\tau ;\theta )d\tau +\sigma_{B}B(t)$$

where the stochastic process is driven by a standard Brown Motion B(t) with a nonlinear drift term $\int_{0}^{t}g(\tau ;\theta )d\tau$ to characterize the nonlinearity in tool wear process. $\sigma_{B}$ is the diffusion coefficient, which represents the inherent temporal variability of wear path. Typically, the formulation of the drift term, which describes the individual variability of different machining conditions, can be expressed as $g(t;\theta )=abt^{b-1}$, g(t;θ)=abexp(bt) or $g(t;\theta )=abt^{b-1}+cd\exp(dt)$. In the proposed model, the nonlinear drift terms were set with different laws in each wear stages. Combining the segmented model in Equation (1) and Wiener process in Equation (2), the proposed three-stage Wiener-process-based model can be formulated as follows:

(3) $$S_{k}=S_{k-1}+u(t_{k};\theta )-u(t_{k-1};\theta )+\sigma_{B}\sqrt{\Delta t}\omega_{k}$$

(4) $$u(t;\theta )=\int_{0}^{t}g(\tau ;\theta )d\tau =at^{b}+c\exp(dt)-c$$

where $\omega_{k}∼N(0,1)$, and a,b,c,d are the parameters of drift term, and satisfied the follow constraints:

(5) $$\left\{\begin{array}{l} c=0,d=0,\;\;t<\tau_{1}\\ a=0,b=0,\;\;t>\tau_{2} \end{array}\right.$$

To define the lifetime and predict the RUL, a concept of first hitting time (FHT) is adopted. According to the concept of FHT, the lifetime T can be defined as:

(6) $$T=\inf\left\{t:S(t)\geq \omega \left|S(0)\right.<\omega \right\}$$

where ω is the failure threshold of the cutting tool. The probability density function (PDF) of lifetime T defined by Equation (6) can be obtained as follows:

(7) $$f_{T}(t)≅\frac{1}{\sqrt{2\pi t}}[\frac{I_{B}(t)}{t}+\frac{g(t;\theta )}{\sigma_{B}}]\exp\left[-\frac{I_{B}^{2}(t)}{2t}\right]$$

where $I_{B}(t)=(\omega -\int_{0}^{t}g(\tau ;\theta )d\tau )/\sigma_{B}$. The RUL $L_{k}$ at time $t_{k}$ can be obtained through time and failure threshold translation and the formulation can be expressed as follows:

(8) $$L_{k}=\inf\left\{l_{k}>0:S(l_{k}+t_{k})\geq \omega \right\}$$

Usually, the degradation state of a cutting tool cannot be measured directly, but formulated with the health indicator (HI) generated from the observable sensor data. The relationship between degradation state and HI can be expressed as follows:

(9) O(t)=ϕ(S(t);ξ)+ε(t)

where O(t) is HI, and ε(t) is the random measurement error with $\varepsilon (t)\in N(0,\gamma^{2})$, which describes the effect of measurement variability of sensing. ϕ(S(t);ξ) denotes the relationship between HI and underlying degradation states, which can be formed as:

(10) $$\phi (S(t);\xi )=\beta_{0}+\beta_{1}S(t)$$

where $\beta_{0}$ and $\beta_{1}$ are the parameters of the linear model. To estimate the underlying degradation state $S_{k}$, the Kalman filter and Rauch–Tung–Striebel smoother (RTS) are introduced into the proposed model. Table 1 shows the steps of Kalman filter algorithm.

After the Kalman filter algorithm, RTS is used to obtain the optimal estimation of the preceding conditional expectations. The smoothing algorithm is summarized as follows in Table 2.

## 3. RUL Prediction Framework

**Figure 2:**
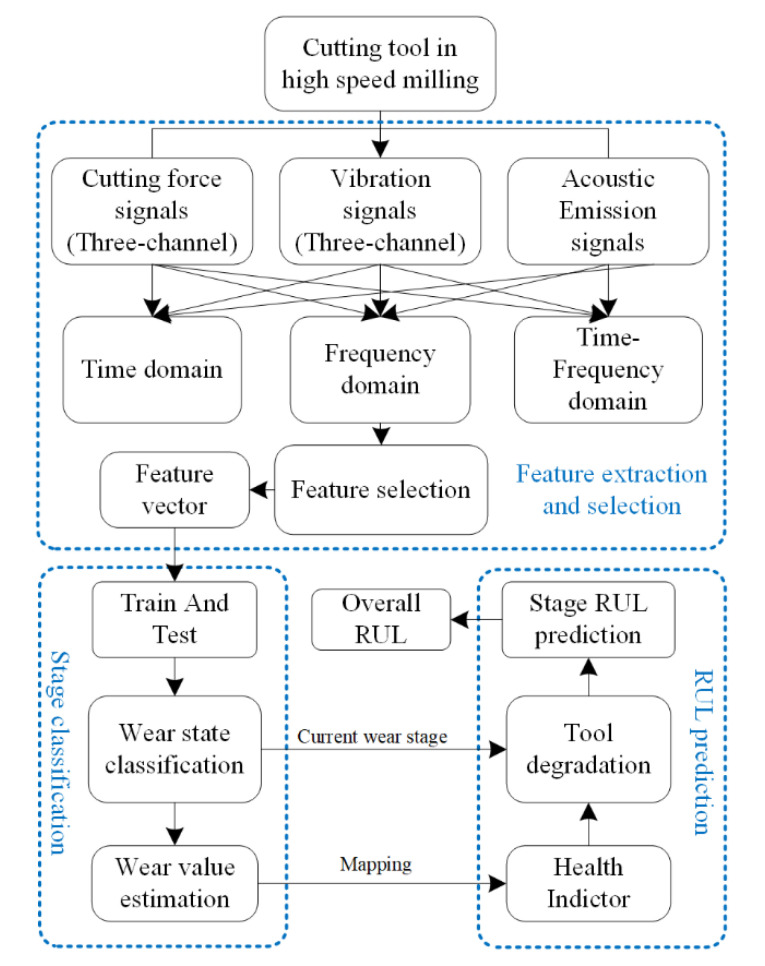
Overall architecture of RUL prediction framework.

The RUL prediction framework is presented in this section and the overall architecture of this framework is depicted in Figure 2. As it can be seen in Figure 2, the time domain, frequency domain and time–frequency domain features are extracted from raw sensor data (i.e., AE, cutting force, vibration) and then SDAE is utilized to automatically select the most relevant features. Next, feature vector is set into ELM to classify tool wear stages and estimate the corresponding wear value. After that, the degradation process of each wear stage is established based on Wiener process and the estimated wear value is regarded as the observable health indicator. At last, the overall useful life can be estimated with the stage RUL prediction.

### 3.1. Feature Extraction and Selection

#### 3.1.1. Feature Extraction

Feature extraction is conducted to reduce noise interference and remove irrelevant signals from the original signals. Statistical features in the time domain and frequency domain are extracted from force signals, vibration signals in each direction (X, Y and Z) and acoustic emission signals. In total, 16 kinds of time domain features namely mean, standard deviation, variance, peak value, peak to peak, root mean square error (RMSE), skewness, kurtosis, kurtosis factor, average absolute value, shape factor, crest factor, impact factor, margin factor, skewness factor and bias factor are extracted from seven channels, respectively. A total of 112 features can be obtained and these features are listed in Table 3.

Eight frequency domain features are also extracted from raw sensor signals. These statistical features are listed in Table 4. In Table 4, $p_{i}$ represents the ith spectrum of the sensor signal f(t), n is the number of spectra and $f_{i}$ is the frequency of the ith spectrum.

The wavelet packet decomposition (WP) is a kind of time–frequency domain analysis tool and is propitious to analyze the non-stationary and time-varying signal. It can decompose the raw signal into multiple levels and each level consists of several frequency bands, in which abundant information can be obtained. In this study, three channels of force, three channels of vibration and one channel acoustic emission signals are decomposed into seven levels by WP. The energy of a signal can be expressed as Equation (11), in which $t_{k}$ is the wavelet packet coefficient [23]. The summary energy of all frequency bands at the jth level can be expressed as Equation (12). Furthermore, the normalization of energy can be expressed as Equation (13). According to Equation (13), seven time–frequency domain features can be obtained in total.

(11) $$E_{j,n}=\sum_{k}{\left|c_{j,n,k}\right|}^{2}$$

(12) $$E_{tot}=\sum_{n}E_{j,n}$$

(13) $$P_{j,n}=\frac{E_{j,n}}{E_{tot}}$$

#### 3.1.2. Automatic Feature Selection with SDAE

In total, 175 features are extracted in Section 3.1.1, which contain redundancy and cross-correlation information. The proposed model would go through a curse of dimensionality if all the 175 features participated in the calculation. Moreover, the high-dimensionality features would increase the computational cost and cause the unstable results of recognition. To deal with these problems, SDAE is utilized for the feature dimensionality reduction in this study, which is composed of two DAEs. The raw features with high dimensionality are used as the input in the first DAE. The low-dimensional and representative features can be obtained robustly based on the hidden layer of the second DAE. To employ the SDAE for feature dimensionality reduction, the relevant parameters are set as follows: learning rate, 1.0; sparsity penalty, 0.05; activation function, sigmoid; input zero masked fraction, 0.5. In this study, to obtain the most representative features, the selection of node sizes in the SDAE is researched. The node number of the hidden layer is chosen from 10 to 160 with the interval of 10 in the first DAE and from 5 to 50 with the interval of 10 in the second DAE, respectively. Considering the computation time and robustness, we finally recommend the node sizes to be 30 and 15 in the first and second DAE, respectively.

### 3.2. Tool Wear Stage Classification and Health Indicator Construction

Current wear stages are determined and health indictor is constructed based on the feature vector. Conventionally, this step can be conducted with a machine-learning-based or deep-learning-based algorithm. In this subsection, we adopt ELM to perform the classification and regression task, while other intelligent algorithms (e.g., SVM, BP Network, RVM, etc.) can also be used as an alternative. 

ELM was first proposed by Huang in 2006 [24] and then widely used for classification problems. However, the abovementioned method is only suitable for binary classification, while the tool wear stages in this study are more than two. To address this problem, pairwise coupling was utilized to estimate the probabilistic outputs of multi-class [25]. Suppose that there are C different wear states, then the multi classification problem is transformed to C(C−1)/2 binary classification problems. For the cth binary classification problem, the probability of i class and j class can be written as:

(14) $$r_{ij}=P\left(t=i\left|t=i\;or\;j,x\right.\right)$$

where P(.|.) is the probability output of ELM with sigmoid function. By fusing the probabilities of C(C−1)/2 binary classification problems, the probability of i class in multi classification can be expressed as:

(15) $$p_{i}=P\left(t=i|x\right),i=1,2,\cdots ,C$$

Then the probability $p_{i}$ can be obtained by solving the following objective function:

(16) $$\underset{P}{\min}\sum_{i=1}^{C}\sum_{j:j\neq i}^{C}{\left(r_{ji}p_{i}-r_{ij}p_{j}\right)}^{2}s.t.\sum_{i}^{C}p_{i}=1$$

The objective function of Equation (16) can be rewritten as

(17) $$\underset{P}{\min}2P^{T}QP\equiv \underset{P}{\min}\frac{1}{2}P^{T}QP$$

where

(18) $$Q_{ij}=\left\{\begin{array}{l} \sum_{s:s\neq i}r_{si}^{2},\;\;\;i=j\\ -r_{ji}r_{ij},\;\;i\neq j \end{array}\right.$$

Finally, probabilistic outputs $P=\{p_{1},p_{1},\cdots ,p_{C}\}$ can be solved by the following matrix

(19) $$\left[\begin{array}{cc} Q & e\\ e^{T} & 0 \end{array}\right]\left[\begin{array}{c} P\\ b \end{array}\right]=\left[\begin{array}{c} 0\\ 1 \end{array}\right]$$

where b is the Lagrangian multiplier of the equality constraint in Equation (16), e is the C×1 vector of all ones and 0 is the C×1 vector of all zeros. By ranking the probabilistic outputs, we can obtain the current wear stage. After classifying the tool wear stage, the tool wear value can be estimated with regression ability of ELM. Moreover, the estimated tool wear value can be used as the observable health indictor.

### 3.3. Parameter Estimation and RUL Prediction

In this subsection, the initial parameters of the proposed model are first estimated with the historical sensor data and wear label and then updated online with the new sensory data. Benefit from the offline estimation, the parameters that update by our algorithm can converge quickly and obtain remarkable performances.

#### 3.3.1. Offline Estimation of Initial Model Parameters

According to the proposed three-stage mode in Section 2, we can obtain the state-space model of wear process:

(20) $$\left\{\begin{array}{l} S_{k}=S_{k-1}+u(t_{k};\theta )-u(t_{k-1};\theta )+\sigma_{B}\sqrt{\Delta t}\omega_{k}\\ O_{k}=\beta_{0}+\beta_{1}S_{k}+\varepsilon_{k} \end{array}\right.$$

Supposing there are N different cutting tools and each cutting tool contained the same measure time in the historical tool wear data. Let $S_{n}={\left[s_{n,1},s_{n,2},\cdots ,s_{n,m}\right]}^{T}$ denotes the nth cutting tool with m wear stages, and the incremental data is $\Delta S_{n}={\left[\Delta s_{n,1},\Delta s_{n,2},\cdots ,\Delta s_{n,m}\right]}^{T}$. Based on the incremental dataset and maximum likelihood estimation (MLE), the log-likelihood function of $\Theta =\left[a,b,c,d,\sigma_{B}\right]$ can be written as follows:

(21) $$\ln(L(\Theta \left|S\right.))=-\frac{NM}{2}\ln(2\pi \Delta t)-\frac{NM}{2}\ln\sigma_{B}^{2}-\frac{1}{2\sigma_{B}^{2}}\sum_{n=1}^{N}\sum_{i=1}^{M}{\left(\Delta s_{n,i}-\left(u\left(t_{i};\theta \right)-u\left(t_{i-1};\theta \right)\right)\right)}^{2}$$

For the convenience of calculation and simplification of expression, we define two intermediate variables $P_{i}$ and $Q_{i}$ as follows:

(22) $$\left\{\begin{array}{l} P_{i}={\left(i\Delta t\right)}^{b}-{\left(\left(i-1\right)\Delta t\right)}^{b}\\ Q_{i}=\exp\left(d\cdot i\Delta t\right)-\exp\left(d\cdot \left(i-1\right)\Delta t\right) \end{array}\right.$$

Then, the log-likelihood function can be expressed as:

(23) $$\ln(L(\Theta \left|S\right.))=-\frac{NM}{2}\ln(2\pi \Delta t)-\frac{NM}{2}\ln\sigma_{B}^{2}-\frac{1}{2\sigma_{B}^{2}\Delta t}\sum_{n=1}^{N}\sum_{i=1}^{M}{\left(\Delta s_{n,i}-\left(aP_{i}+cQ_{i}\right)\right)}^{2}$$

To estimate the parameters Θ, we first fix the parameters b,c,d and then take the partial derivatives of Equation (23) to parameters a and $\sigma_{B}^{2}$.

(24) $$\frac{\partial \ln(L(\theta \left|S\right.))}{\partial a}=-\frac{1}{\sigma_{B}^{2}\Delta t}\sum_{n=1}^{N}\sum_{i=1}^{M}\left(aP_{i}^{2}-\Delta s_{n,i}P_{i}+cP_{i}Q_{i}\right)$$

(25) $$\frac{\partial \ln(L(\theta \left|S\right.))}{\partial \sigma_{B}^{2}}=-\frac{NM}{2\sigma_{B}^{2}}+\frac{1}{2\sigma_{B}^{4}\Delta t}\sum_{n=1}^{N}\sum_{i=1}^{M}{\left(\Delta s_{n,i}-\left(aP_{i}+cQ_{i}\right)\right)}^{2}$$

Then the estimated parameters $\hat{a}$ and ${\hat{\sigma }}_{B}^{2}$ can be obtained by zeroing Equations (24) and (25), respectively.

(26) $$\hat{a}=\frac{\sum_{n=1}^{N}\sum_{i=1}^{M}\left(\Delta s_{n,i}P_{i}-cP_{i}Q_{i}\right)}{\sum_{n=1}^{N}\sum_{i=1}^{M}P_{i}^{2}}$$

(27) $${\hat{\sigma }}_{B}^{2}=\frac{\sum_{n=1}^{N}\sum_{i=1}^{M}{\left(\Delta s_{n,i}-\left(\hat{a}P_{i}+cQ_{i}\right)\right)}^{2}}{NM\Delta t}$$

The log-likelihood function of parameters b,c,d can be deduced by taking Equations (26) and (27) into Equation (23), and the parameters b,c,d can be calculated by maximizing Equation (28) with a multidimensional search algorithm.

(28) $$\ln(L(\Theta \left|S\right.))=-\frac{NM}{2}\ln(2\pi \Delta t)-\frac{NM}{2}\ln{\hat{\sigma }}_{B}^{2}-\frac{NM}{2}$$

Furthermore, the estimation parameters a and $\sigma_{B}^{2}$ can be obtained by substituting the estimated parameters b,c,d into Equations (26) and (27), respectively.

#### 3.3.2. Online Updating of Model Parameters

$\Phi =[\Phi_{1},\Phi_{2}]$ is the model parameter vector that need to be updated online with the real-time wear data. The parameter vector consists of two parts, i.e., $\Phi_{1}=[a,b,c,d,\sigma_{B}{]}^{T}$ and $\Phi_{2}=[\beta_{0},\beta_{1},\gamma {]}^{T}$, corresponding to the state equation and observation equation in the state-space model, respectively. In this study, the EM algorithm is used to estimate the parameter vector online because the underlying wear states of the cutting tool cannot be observed directly. It is worth mentioning that the parameters estimated based on the historical tool wear data in Section 3.3.1 are used as the initial parameters in the online updating of model parameters. According to the Bayesian chain principle, the log-likelihood function of underlying wear state $S_{1:k}$ and the wear indicator $O_{1:k}$ constructed by the observable sensor data can be given by:

(29) $$\begin{array}{ll} L\left(S_{1:k},O_{1:k}\left|\Phi \right.\right) & =\ln p(S_{1:k},O_{1:k}\left|\Phi \right.)=\ln\left[\prod_{i=1}^{k}p\left(S_{i}\left|S_{i-1};\Phi \right.\right)\left|\prod_{i=1}^{k}p\left(O_{i}\left|S_{i};\Phi \right.\right)\right.\right]\\ & =-k\ln\left(2\pi \sqrt{\Delta t}\right)-k\ln\gamma -\frac{1}{2\gamma^{2}}\sum_{i=1}^{k}{\left[O_{i}-\phi (S_{k};\xi )\right]}^{2}-\\ & \;k\ln\sigma_{B}-\frac{1}{2\sigma_{B}^{2}\Delta t}\sum_{i=1}^{k}\left[S_{k}-S_{k-1}-\left(u\left(t_{i};\theta \right)-u\left(t_{i-1};\theta \right)\right)\right]{}^{2} \end{array}$$

Substituting the model parameters and omitting the variables that are unrelated to the estimated parameters, we can obtain the expectation of log-likelihood function.

(30) $$\begin{array}{cc} \Psi \left(\Phi ,{\hat{\Phi }}^{\left(j\right)}\right) & ={Ε}_{\Phi^{\left(j\right)}}\left(L\left(S_{1:k},O_{1:k}\left|\Phi \right.\right)\left|O_{1:k}\right.\right)\\ & \propto -k\ln\sigma_{B}-k\ln\gamma -\frac{1}{2\gamma^{2}}\sum_{i=1}^{k}{Ε}_{{\hat{\Phi }}^{\left(j\right)}}\left\{{\left[O_{i}-\phi (S_{k};\xi )\right]}^{2}\left|O_{1:k}\right.\right\}-\\ & \;\frac{1}{2\sigma_{B}^{2}\Delta t}\sum_{i=1}^{k}{Ε}_{{\hat{\Phi }}^{\left(j\right)}}\left\{{\left[S_{k}-S_{k-1}-\left(u\left(t_{i};\theta \right)-u\left(t_{i-1};\theta \right)\right)\right]}^{2}\left|O_{1:k}\right.\right\} \end{array}$$

Then, the estimation procedure consists of two steps: expectation step (E-step) and maximization step (M-step).

- **E-Step:** Calculating the expectation of log-likelihood function based on the jth iteration.

(31) $$\begin{array}{c} \Psi \left(\Phi ,{\hat{\Phi }}^{\left(j\right)}\right)\propto -k\ln\sigma_{B}-k\ln\gamma -\frac{1}{2\gamma^{2}}\sum_{i=1}^{k}\left(O_{i}^{2}+P_{i\left|k\right.}+{\hat{S}}_{i\left|k\right.}^{2}-2O_{i}{\hat{S}}_{i\left|k\right.}\right)-\\ \;\frac{1}{2\sigma_{B}^{2}\Delta t}\sum_{i=1}^{k}\left[A_{i}-2aP_{i}B_{i}-2cQ_{i}B_{i}+{\left(aP_{i}+cQ_{i}\right)}^{2}\right] \end{array}$$

where the intermediate variables ${\hat{S}}_{i\left|k\right.}$, ${\hat{S}}_{i-1\left|k\right.}$$P_{i\left|k\right.}$, $P_{i-1\left|k\right.}$ and $P_{i,i-1\left|k\right.}$ can be calculated through the RTS algorithm presented in Section 2. The details of these variables are as follows:

(32) $$\left\{\begin{array}{l} {\hat{S}}_{i\left|k\right.}={Ε}_{{\hat{\Phi }}^{\left(j\right)}}\left(S_{i}\left|O_{1:k}\right.\right)={\hat{S}}_{j\left|k\right.}\\ P_{i\left|k\right.}={Ε}_{{\hat{\Phi }}^{\left(j\right)}}\left(S_{i}^{2}\left|O_{1:k}\right.\right)-{\hat{S}}_{i\left|k\right.}=P_{j\left|k\right.}\\ P_{i,i-1\left|k\right.}={Ε}_{{\hat{\Phi }}^{\left(j\right)}}\left(S_{i}S_{i-1}\left|O_{1:k}\right.\right)-{\hat{S}}_{i\left|k\right.}{\hat{S}}_{i-1\left|k\right.}=M_{j\left|k\right.} \end{array}\right.$$

Then $A_{i}$ and $B_{i}$ can be expressed by the above intermediate variables.

(33) $$\left\{\begin{array}{l} A_{i}=P_{i\left|k\right.}+{\hat{S}}_{i\left|k\right.}^{2}+P_{i-1\left|k\right.}+{\hat{S}}_{i-1\left|k\right.}^{2}-2P_{i,i-1\left|k\right.}-2{\hat{S}}_{i\left|k\right.}{\hat{S}}_{i-1\left|k\right.}\\ B_{i}={\hat{S}}_{i\left|k\right.}-{\hat{S}}_{i-1\left|k\right.} \end{array}\right.$$

- **M-Step:** Calculating the parameter ${\hat{\Phi }}_{}^{\left(j+1\right)}$ in the (j+1)th iteration.

(34) $${\hat{\Phi }}_{}^{\left(j+1\right)}=\arg\underset{\Phi }{\max}\left[\Psi \left(\Phi ,{\hat{\Phi }}^{\left(j\right)}\right)\right]$$

Equation (30) can be divided into two parts:

(35) $$\left\{\begin{array}{l} \Psi_{1}\left(\Phi_{1},{\hat{\Phi }}_{1}^{\left(j\right)}\right)\propto -k\ln\sigma_{B}-\frac{1}{2\sigma_{B}^{2}\Delta t}\sum_{i=1}^{k}\left[A_{i}-2aP_{i}B_{i}-2cQ_{i}B_{i}+{\left(aP_{i}+cQ_{i}\right)}^{2}\right]\\ \Psi_{2}\left(\Phi_{2},{\hat{\Phi }}_{2}^{\left(j\right)}\right)\propto -k\ln\gamma -\frac{1}{2\gamma^{2}}\sum_{i=1}^{k}\left(O_{i}^{2}+P_{i\left|k\right.}+{\hat{S}}_{i\left|k\right.}^{2}-2O_{i}{\hat{S}}_{i\left|k\right.}\right) \end{array}\right.$$

The parameters $\Phi_{1}$ and $\Phi_{2}$ can be estimated by using the MLE that was introduced in Section 3.3.1 and the specific expression of these parameters are given by

(36) $$\left\{\begin{array}{l} \hat{a}=\frac{\sum_{i=1}^{k}\left(P_{i}B_{i}-cP_{i}Q_{i}\right)}{\sum_{i=1}^{k}P_{i}^{2}}\\ {\hat{\sigma }}_{B}^{2}=\frac{\sum_{i=1}^{k}\left[A_{i}-2aP_{i}B_{i}-2cQ_{i}B_{i}+{\left(aP_{i}+cQ_{i}\right)}^{2}\right]}{k\cdot \Delta t}\\ {\hat{\gamma }}^{2}=\frac{\sum_{i=1}^{k}\left(O_{i}^{2}+P_{i\left|k\right.}+{\hat{S}}_{i\left|k\right.}^{2}-2O_{i}{\hat{S}}_{i\left|k\right.}\right)}{k} \end{array}\right.$$

#### 3.3.3. RUL Prediction

After the model parameter estimation, the overall lifetime and remaining useful life to a specific moment and their corresponding PDFs can be obtained.

- PDF of lifetime

The PDF of lifetime can be described as follows:

(37) $$\begin{array}{ll} f_{T}\left(t\left|\Theta \right.\right)\approx & \frac{\omega -at^{b}\left(1-b\right)-c\left(\exp\left(d\cdot t\right)-d\cdot t\exp\left(d\cdot t\right)-1\right)}{\sqrt{2\pi \sigma_{B}^{2}t^{3}}}\cdot \\ & \exp\left[-\frac{{\left(\omega -at^{b}-c\exp\left(d\cdot t\right)+c\right)}^{2}}{2\sigma_{B}^{2}t}\right] \end{array}$$

- PDF of RUL up to $t_{k}$

Model parameters, underlying wear state ${\hat{S}}_{k\left|k\right.}$ and its variance $P_{k\left|k\right.}$ can be updated by the EKF once the new observable data are available. With the wear state and updated parameters, the analytical form of the RUL distribution at time $t_{k}$ can be written as Equation (7) based on the law of total probability.

(38) $$\begin{array}{cc} f_{L_{k}}\left(l_{k}|\Theta ,O_{1:k}\right) & \approx \int_{-\infty }^{\infty }f_{L_{k}}\left(l_{k}|\Theta ,S\left(t_{k}\right),O_{1:k}\right)p\left(s_{k}|O_{1:k}\right)ds_{k}\\ & \approx E_{S\left(t_{k}\right)|O_{1:k}}\left\{f_{L_{k}}\left(l_{k}|\Theta ,S\left(t_{k}\right),O_{1:k}\right)\right\}\\ & \approx \frac{1}{\sqrt{2\pi l_{k}^{2}\left(P_{k|k}+\sigma_{B}^{2}l_{k}\right)}}[\omega -v\left(l_{k}\right)+abl_{k}{\left(l_{k}+t_{k}\right)}^{b-1}+cdl_{k}\exp\left(d\cdot \left(l_{k}+t_{k}\right)\right).\\ & -\frac{P_{k|k}\left(\omega -v\left(l_{k}\right)\right)+{\hat{S}}_{k|k}\sigma_{B}^{2}l_{k}}{P_{k|k}+\sigma_{B}^{2}l_{k}}]\exp\left(-\frac{{\left(\omega -v\left(l_{k}\right)-{\hat{S}}_{k|k}\right)}^{2}}{2\left(P_{k|k}+\sigma_{B}^{2}l_{k}\right)}\right) \end{array}$$

where $\nu \left(l_{k}\right)=a{\left(l_{k}+t_{k}\right)}^{b}+c\exp\left(d\cdot \left(l_{k}+t_{k}\right)\right)-at_{k}^{b}-c\exp\left(d\cdot t_{k}\right)$.

## 4. Experimental Study

In this section, the proposed tool wear model is demonstrated with a practical experimental study based on the dataset from the 2010 Prognostic and Health Management (PHM) competition (Prognostics and Health Management Society 2010) [26].

### 4.1. Experiment Set-Up and Data Acquisition

**Figure 3:**
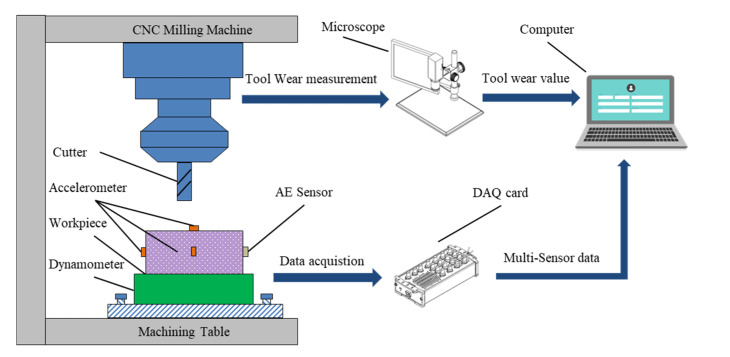
Experimental set-up.

**Figure 4:**
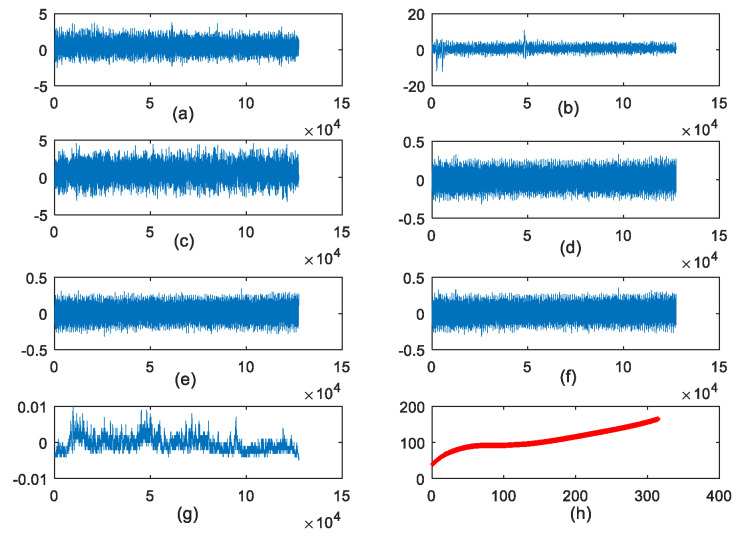
Raw data of Cutter #1. (**a**) Vibration signal in X direction; (**b**) Vibration signal in Y direction; (**c**) Vibration signal in Z direction; (**d**) Cutting force signal in X direction; (**e**) Cutting force signal in Y direction; (**f**) Cutting force signal in Z direction; (**g**) Acoustic emission signal; (**h**) True wear value.

In the conducted experiment as shown in Figure 3, a high-speed CNC milling machine with a three-flute ball-nose cutter was used to mill the workpieces (material: Inconel 718). During the cutting process, the spindle rotation speed was set as 10,400 r/min and the feed rate was set as 1555 mm/min, while the cutting width of Y direction (radial) and the cutting depth of Z direction (axial) were set as 0.125 mm and 0.2 mm, respectively. A quartz three-channel dynamometer was mounted on the CNC milling machine to measure the cutting force. Simultaneously, vibration signal along the same three directions (x, y and z) was measured by a piezo-accelerometer. Moreover, an acoustic emission sensor was mounted on the workpiece to capture the high-frequency stress wave generated by the cutting process. All the signals collected above were amplified by the charge amplifier and eventually converted into voltage signals. Then, the voltage signal of seven channels was sampled with 50 kHz. Additionally, the explicit tool wear value was measured by the microscope in an offline way after each cutting. Finally, three groups of experiments (Cutter #1, Cutter #4 and Cutter #6) were conducted. Each cutting process lasted for 315 passes and each cutting pass was four seconds. The raw data consist of two parts: true wear value measured with a microscope and several channels’ sensory data. Figure 4 depicts the several channels’ sensory data at a cutting pass and the true wear value of the entire life of Cutter #1.

### 4.2. Feature Selection with SDAE

**Figure 5:**
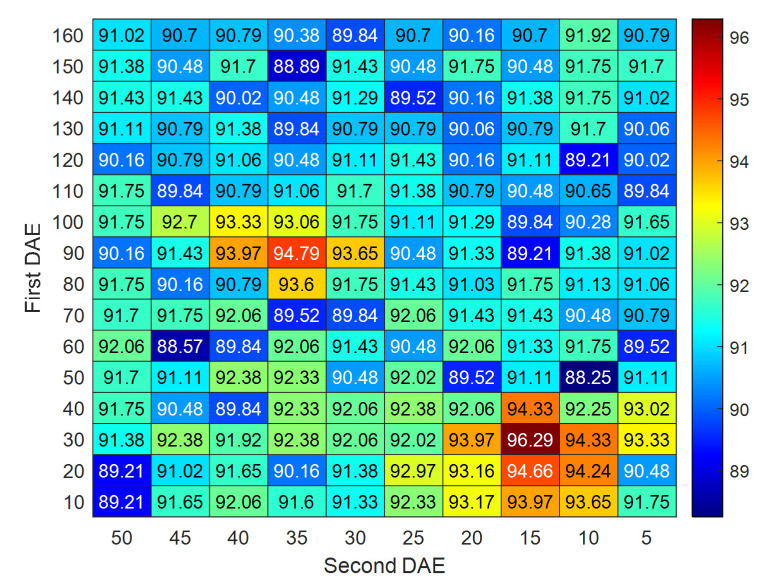
Pre-classify accuracy rate with different node sizes of SDAE.

In this study, to obtain the most representative features, the selection of node sizes in the SDAE was researched. The node number of the hidden layer was chosen from 10 to 160 with the interval of 10 in the first DAE and from 5 to 50 with the interval of 10 in the second DAE, respectively. Given different combinations of node sizes, the pre-classify accuracy results can be obtained and presented in Figure 5. As seen in Figure 5, the classification accuracy rate can approach the maximum when the nodes sizes in the first and second DAE were set at 30 and 15, respectively. It can be also observed from Figure 5 that the accuracy rate is relatively high when the nodes sizes in the first and second DAE were set at 90 and 35. However, more features will improve computing time. Thus, the node sizes in the hidden layers of the first and second DAE were recommended to be 30 and 15, respectively.

### 4.3. Experimental Results

In this study, each group of experiment data describe the whole wear process of a cutter and can be divided into three stages according the to the Taylor tool life curve [12]. We randomly select two groups of experiment data as the training set and the left one as a testing set. Therefore, we can obtain three groups of validation results (i.e., Cutter #1, Cutter #4, and Cutter #6). In order to compare the effectiveness of the proposed model, three other models (i.e., M1 [17], M2 [27] and M3 [28]) from previous studies, which follow the power law, exponential law and an integrated power–exponential law, respectively, are also conducted with the same date. 

#### 4.3.1. Model Parameter Estimation

Parameters of the proposed three-stage Wiener-process-based model were estimated with the experimental data. The key model parameters of each stage are list in Table 5. According to the proposed model in Section 2, the three stage list in Table 5 can be expressed as follows:

(1) **Stage-1**
(39) $$u(t;\theta )=at^{b}$$
(2) **Stage-2**
(40) $$u(t;\theta )=at^{b}+c\exp(dt)-c$$
(3) **Stage-3**
(41) u(t;θ)=cexp(dt)−c

**Table 5:** Estimated results of the model parameters.

Model Parameters	Cutter #1			Cutter #4			Cutter #6		
	Stage-1	Stage-2	Stage-3	Stage-1	Stage-2	Stage-3	Stage-1	Stage-2	Stage-3
a	31.7602	89.7611	―	23.4435	52.1428	―	38.1735	89.0511	―
b	0.2582	0.0001	―	0.3071	0.1082	―	0.2291	0.0462	―
c	―	3.9420	122.8121	―	3.0810	89.6324	―	50.1321	128.2356
d	―	0.0144	0.0031	―	0.0082	0.0073	―	0.0019	0.0044
$\beta_{0}$	0.0107	0.0969	0.0925	0.0108	0.0145	0.0283	0.0028	0.0173	0.0543
$\beta_{1}$	0.9837	1.1215	0.9995	1.0031	0.9998	0.9999	1.0001	0.9986	0.9998
$\sigma_{B}$	0.5046	0.1312	0.4480	0.4612	0.1678	0.9885	0.9715	0.1150	0.7297
γ	0.0140	0.0133	0.0436	0.0135	0.0138	0.0324	0.0145	0.0135	0.0397

#### 4.3.2. RUL Prediction Result

**Figure 6:**
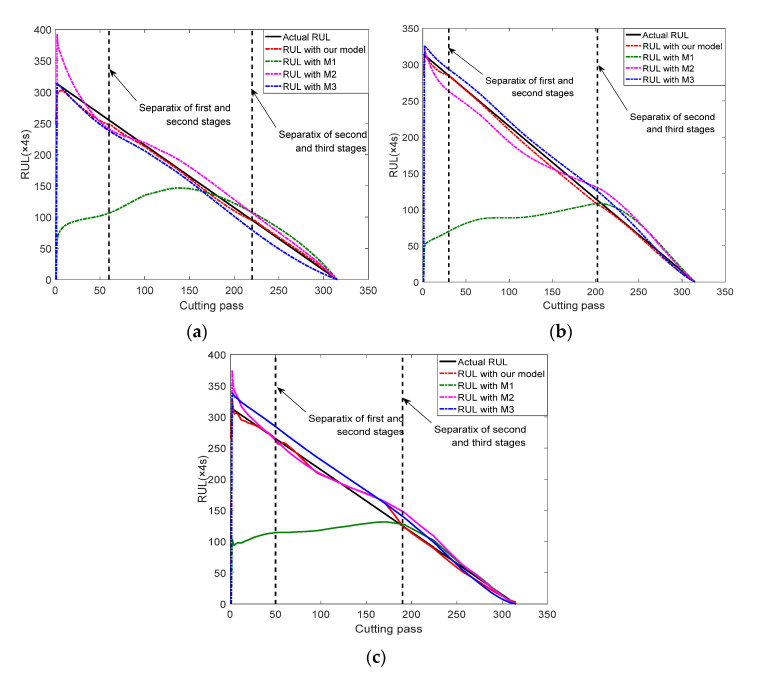
RUL prediction results with proposed model and other existing models. (**a**) Cutter #1; (**b**) Cutter #4; (**c**) Cutter #6.

The whole life of each cutter is 315 cutting passes and each cutting pass lasts four seconds. RUL prediction result of three cutters with our proposed model and three other models are present in Figure 6. The prediction results with the proposed three-stage model are much closer to the actual RUL in every wear stage than in the other three models. The performances of the other three models vary in different wear stages. For example, M1 model, built based on the power law, provides poor performances at the first and second stages in both three cutters. Both M2 and M3 models perform better than M1 and present different deviations at the three stages, but there is still a certain gap compared with our three-stage model. These results indicate that our model can accurately predict the RUL of a cutting tool.

**Figure 7:**
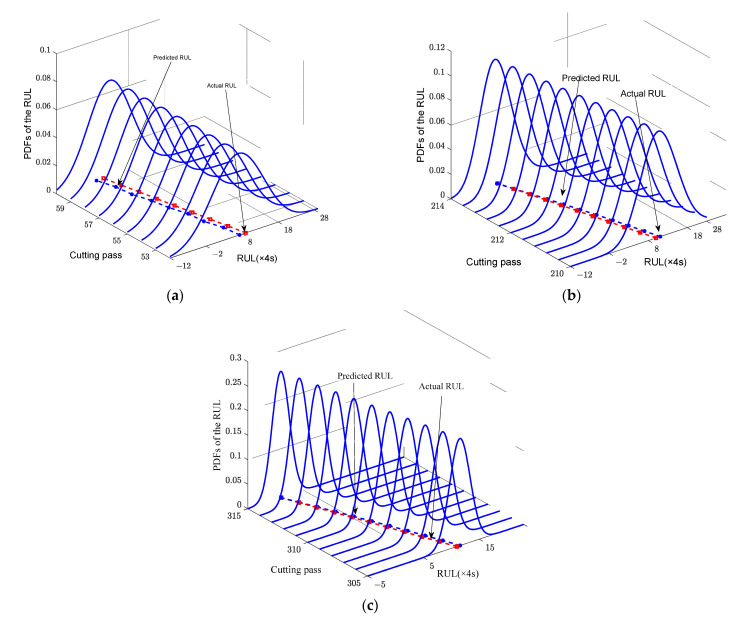
PDFs of Cutter #1 with the proposed model. (**a**) Stage 1; (**b**) Stage 2; (**c**) Stage 3.

To further investigate the superiority of the proposed three-stage Wiener-process-based degradation model, the PDFs of RUL at different wear stages are calculated. Take Cutter #1 as an example, Figure 7 presents the PDFs of RUL, the prediction RULs and actual RULs over a period of cutting passes at three wear stages. As seen in Figure 7, the predicted RULs are closed to the actual RULs and the PDFs calculated with our model can cover the actual RULs at all the three stages.

#### 4.3.3. Comparison and Evaluation

**Figure 8:**
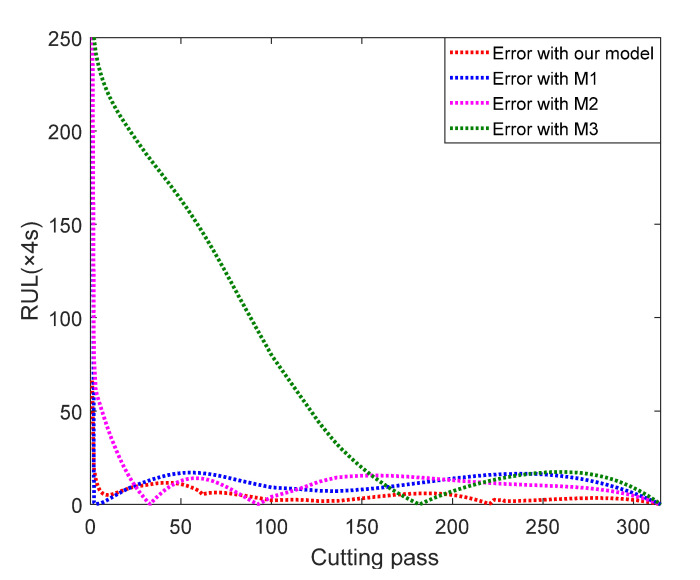
Comparison of RUL prediction errors between our model and other existing models.

In order to compare the proposed three-stage model with other models more clearly, the prediction errors are calculated and the error result of Cutter #1 is present in Figure 8. We can observe that the prediction errors using our model keep in a low range throughout the whole process, while the other three models fluctuate greatly.

To quantitatively compare the RUL prediction accuracy between the proposed model and the other three models, we list the mean square error (MSE), mean absolute error (MAE) and coefficient of determination ($R^{2}$) of estimation error in Table 5. Formulations to calculate the prediction error are defined as follows:

(1) MSE
  (42) $$MSE=\frac{1}{K}\sum_{k=1}^{K}{\left(l_{k}-{\tilde{l}}_{k}\right)}^{2}$$
(2) MAE
  (43) $$MAE=\frac{1}{K}\sum_{k=1}^{K}\left|l_{k}-{\tilde{l}}_{k}\right|$$
(3) $R^{2}$
  (44) $$R^{2}=1-\frac{\sum_{k=1}^{K}{\left(l_{k}-{\tilde{l}}_{k}\right)}^{2}}{\sum_{k=1}^{K}{\left(l_{k}-\bar{l}\right)}^{2}}$$
  where $l_{k}$, ${\tilde{l}}_{k}$ and $\bar{l}$ denote the predicted RUL, actual RUL and mean actual RUL, respectively. We can observe from Table 6 that our model achieves much better performance than the other three models.

**Figure 9:**
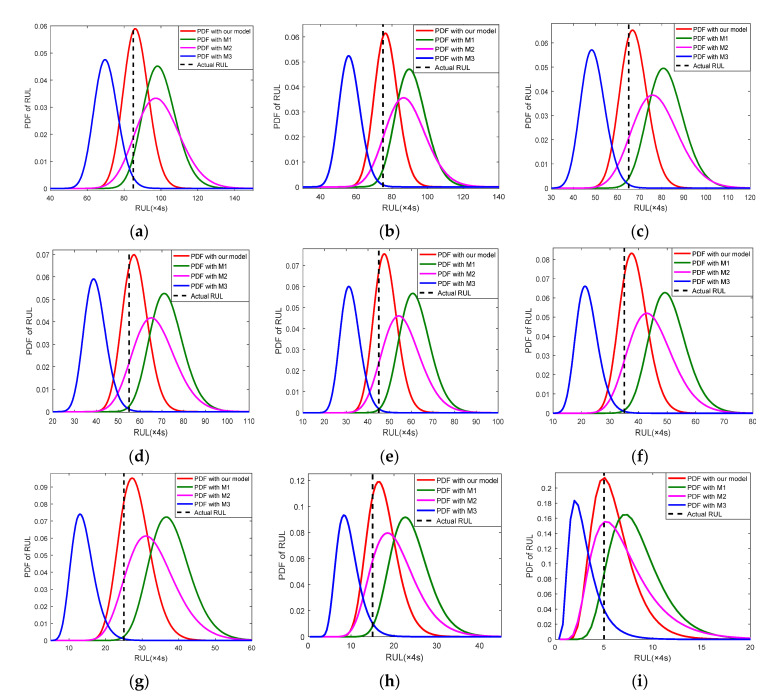
Comparison of RULs and estimation errors between our model and power model. (**a**) Cutting pass 230; (**b**) Cutting pass 240; (**c**) Cutting pass 250; (**d**) Cutting pass 260; (**e**) Cutting pass 270; (**f**) Cutting pass 280; (**g**) Cutting pass 290; (**h**) Cutting pass 300; (**i**) Cutting pass 310.

To further explore the inherent reasons for the performance gap between the proposed model and the other three models, a comparative study is carried out. PDFs of Cutter #1 were calculated with different models. Figure 9 presents the PDFs of different models with an interval of ten cutting passes from the 230 to 310 cutting passes. It can be seen from Figure 9 that the calculated PDFs using our model are much tighter and higher than the other three models for all the nine cutting passes, which indicates that our model can reduce the uncertainty of RUL prediction when compared with other existing models. Additionally, the estimated RULs are more and more close to the true RUL with the accumulation of sensor data. All these results indicate that the parameters of our model are updated constantly with online sensing data, which causes the proposed model to become more similar to the true degradation model.

## 5. Discussion and Conclusions

This study proposes a three-stage Wiener-process-based degradation model for the cutting tool wear and remaining useful life prediction. Taking full advantage of the Wiener process to describe the degradation processes and ELM to classify tool wear stages, joint implementation of tool wear stage classification and RUL prediction is carried out. Moreover, considering multi-source variabilities in the modeling process of each stage, the proposed model can describe the physical behavior of the tool wear process more precisely and significantly improve the accuracy of RUL prediction. The numerical results obtained using extensive experiments indicate that the proposed model can effectively predict the tool’s remaining useful life in high-speed milling processes. Empirical comparisons show that the proposed three-stage Wiener-process-based model performs better compared to existing models in predicting the tool’s RUL while also providing the analytical form of the RUL distribution that is very useful for the health management of the cutting tool.

Although the experimental studies in this study show that our model can accurately predict the RUL and estimate the wear value of the cutting tool, there are still several issues that need to be studies further. Firstly, the three stages of a cutting tool are assumed mutually independent, which leads to the saltatory prediction result at the boundary of different stages. Therefore, the correlation between different stages needs to be considered. Secondly, we mainly concentrate on the gradual and continuous wear process of a cutting tool; however, the sudden failures of the cutting tool (such as worn, tipping and so on) also need to be considered in the practical application. It is worth pursuing this research direction in our future work.

## Figures and Tables

**Table 1:** Computational flow of Kalman filter algorithm.

**Step 1:** Set the parameters $\theta ,\sigma_{B},\xi ,\gamma$
**Step 2:** Estimate the state ${\hat{S}}_{k\left|k-1\right.}$ and variance $P_{k\left|k-1\right.}$ ${\hat{S}}_{k\left|k-1\right.}={\hat{S}}_{k-1\left|k-1\right.}+\int_{0}^{t_{k}}g(\tau ;\theta )d\tau -\int_{0}^{t_{k-1}}g(\tau ;\theta )d\tau$ $P_{k\left|k-1\right.}=P_{k-1\left|k-1\right.}+\sigma_{B}^{2}\Delta t$
**Step 3:** Calculate the Kalman coefficient K(k) $K(k)=P_{k\left|k-1\right.}/(P_{k\left|k-1\right.}+\gamma^{2})$
**Step 4:** Update the state and variance ${\hat{S}}_{k\left|k\right.}={\hat{S}}_{k\left|k-1\right.}+K(k)(O_{k}-\phi (S_{k};\xi ))$ $P_{k\left|k\right.}=(1-K(k))P_{k\left|k-1\right.}$

**Table 2:** Computational flow of RTS smoothing algorithm.

**Step 1:** Forward iteration through Kalman filter and obtain the optimal estimation ${\hat{S}}_{k\left|k\right.}$ and $P_{k\left|k\right.}$.
**Step 2:** Optimal smoothing estimation of backward iteration $R_{j}=P_{j\left|j\right.}P_{j+1\left|j\right.}^{-1}$ ${\hat{S}}_{j\left|k\right.}={\hat{S}}_{j}+R_{j}({\hat{S}}_{j+1\left|k\right.}-{\hat{S}}_{j+1\left|j\right.})={\hat{S}}_{j}+R_{j}({\hat{S}}_{j+1\left|k\right.}-{\hat{S}}_{j})$ $P_{j\left|k\right.}=P_{j\left|j\right.}+R_{j}^{2}(P_{j+1\left|k\right.}-P_{j+1\left|j\right.})$
**Step 3:** Initialization
$M_{k\left|k\right.}=\left(1-K_{k}\Delta t\right)P_{k-1\left|k-1\right.}$
**Step 4:** Smoothing covariance calculation of backward iteration $M_{j\left|k\right.}=P_{j\left|j\right.}S_{j-1}+S_{j}(M_{j+1\left|k\right.}-P_{j\left|j\right.})S_{j-1}$

**Table 3:** Statistical features in the time domain.

Feature	Expression	Feature	Expression
T1	$M=\frac{1}{T}\sum_{t=1}^{T}f(t)$	T9	$KR=\frac{\sum_{t=1}^{T}f{\left(t\right)}^{4}}{\sqrt{\sum_{t=1}^{T}f{\left(t\right)}^{2}}}$
T2	$\mathrm{STD}=\sqrt{\frac{1}{T}\sum_{t=1}^{T}{\left(f(t)-M\right)}^{2}}$	T10	$ARV=\frac{1}{T}\sum_{t=1}^{T}\left|f(t)\right|$
T3	$VAR=\frac{1}{T}\sum_{t=1}^{T}{\left(f(t)-M\right)}^{{}^{2}}$	T11	$\mathrm{SF}=\frac{RMS}{ARV}$
T4	P=max(f(t))	T12	$CP=\frac{PP}{\mathrm{RMS}}$
T5	PP=max(f(t))−min(f(t))	T13	$\mathrm{IF}=\frac{\mathrm{PP}}{ARV}$
T6	$\mathrm{RMS}=\sqrt{\frac{1}{T}\sum_{t=1}^{T}{\left(f(t)\right)}^{2}}$	T14	$CL=\frac{\left|PP\right|}{{\left(\frac{1}{T}\sqrt{\sum_{t=1}^{T}\left|f(t)\right|}\right)}^{2}}$
T7	$SK=\frac{\frac{1}{T}\sum_{t=1}^{T}{\left(f(t)-M\right)}^{3}}{STD^{3}}$	T15	$CS=\frac{\sum_{t=1}^{T}f{\left(t\right)}^{3}}{M}$
T8	$\mathrm{KU}=\frac{\frac{1}{T}\sum_{t=1}^{T}{\left(f(t)-M\right)}^{4}}{STD^{4}}$	T16	$CK=\frac{\sum_{t=1}^{T}f{\left(t\right)}^{4}}{M}$

**Table 4:** Statistical features in the frequency domain.

Feature	Expression	Feature	Expression
F1	$PC=\sum_{i=1}^{n}p_{i}{}^{2}$	F5	$M_{p}=\frac{1}{n}\sum_{i=1}^{n}S{\left(f\right)}_{i}$
F2	$FC=\frac{\sum_{i=1}^{n}f_{i}p_{i}}{\sum_{i=1}^{n}p_{i}}$	F6	$VAR_{p}=\frac{\sum_{i=1}^{n}{\left(S{\left(f\right)}_{i}-M_{p}\right)}^{2}}{n-1}$
F3	$VF=\frac{\sum_{i=1}^{n}{\left(f_{i}-FC\right)}^{2}p_{i}}{\sum_{i=1}^{n}p_{i}}$	F7	KUp=1n∑i=1n(S(f)i−Mp)4VARp42
F4	$MSF=\frac{\sum_{i=1}^{n}f_{i}{}^{2}p_{i}}{\sum_{i=1}^{n}p_{i}}$	F8	SKp=1n∑i=1n(S(f)i−Mp)3VARp32

**Table 6:** RUL prediction results of the different models.

Model	Cutter #1		Cutter #4		Cutter #6	
	MSE	MAPE (%)	MSE	MAPE (%)	MSE	MAPE (%)
Our model	42.80	4.78	22.76	5.19	48.64	7.01
M1	9355.50	35.85	1367.30	41.99	8867.40	28.69
M2	492.39	11.85	569.57	12.66	462.39	9.20
M3	457.76	13.96	382.71	7.99	528.68	13.27

## Data Availability

The data associated with this study are publicly accessible at the Prognostics and Health Management Society website: https://www.kaggle.com/rabahba/phm-data-challenge-2010 (accessed on 13 May 2022).
